# Supplementary material for: Assessment of NK Cell Activity Based on NK Cell-Specific Receptor Synergy in Peripheral Blood Mononuclear Cells and Whole Blood
Source: Int J Mol Sci. 2020 Oct 30;21(21):8112. doi: 10.3390/ijms21218112 (PMC7662667; doi:10.3390/ijms21218112)
Supplement: Supplementary file 1 [file ijms-21-08112-s001.pdf]

Supplemental figures

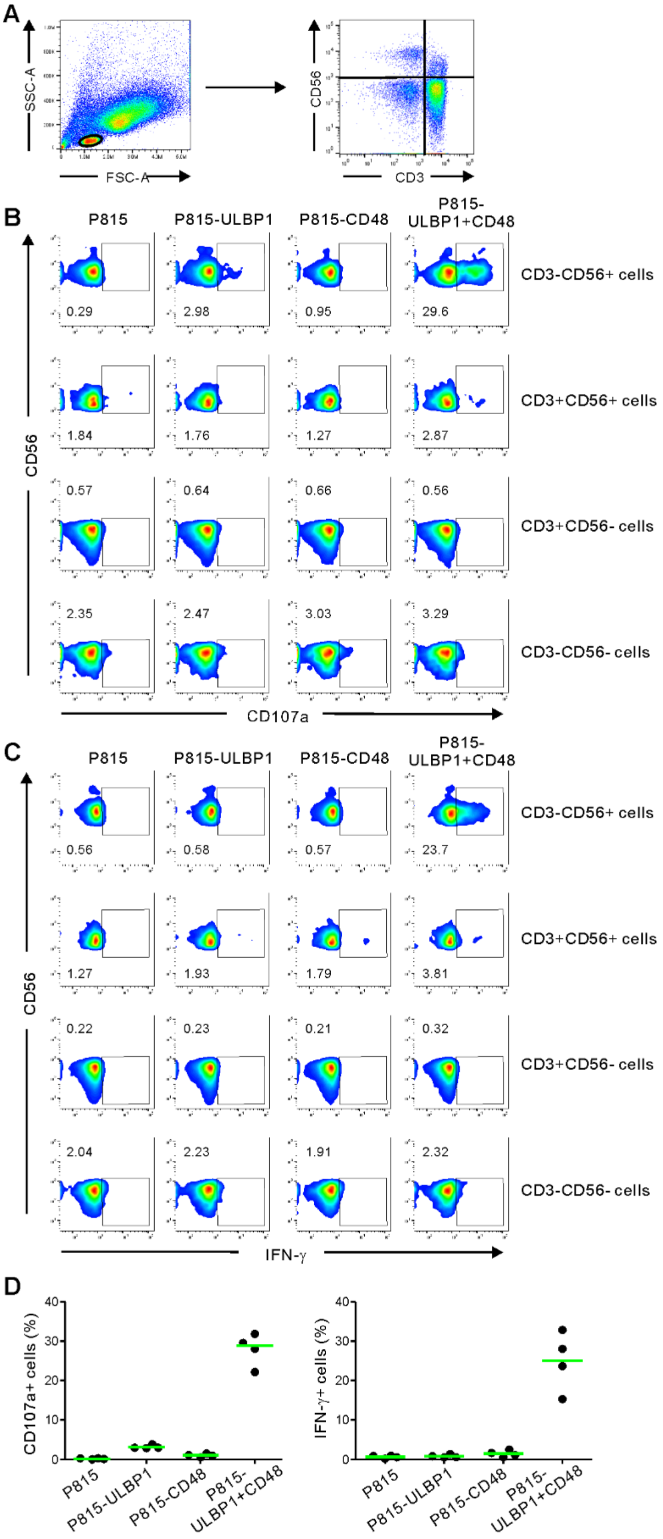

Figure S1. Selective and synergistic stimulation of NK cells by P815-ULBP1+CD48 target cells.

(A) Representative FACS profiles showing the strategy for identifying CD3-CD56+ NK cells within the lymphocyte gate. (B, C) PBMCs from the HD group were mixed with P815 cells expressing ULBP1, a ligand for NKG2D, and/or CD48, a ligand for 2B4 (P815 negative control, P815-ULBP1, P815-CD48, or P815-ULBP1+CD48 cells). Representative FACS profiles showing the percentages of CD107a-positive cells after 2 h of stimulation (B) and the percentages of IFN-γ-positive cells after 6 h of stimulation (C) in the indicated cell populations. (D) Summary graphs showing the percentages of CD107a+ (left) and IFN-γ+ (right) CD3-CD56+ NK cells. Horizontal bars (green) indicate the medians.

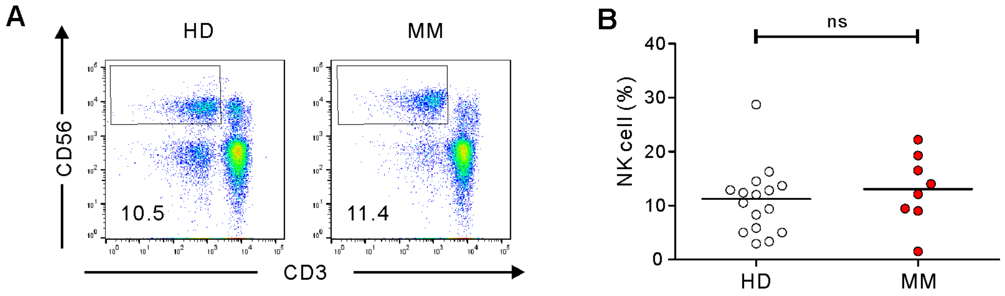

Figure S2. Patients with MM have comparable NK cell frequencies to healthy donors.
(A) Representative FACS profiles showing the frequency of CD3-CD56+ NK cells. (B) The percentages of total NK cells in the PBMCs from the HD group (*n* = 16) and MM group (*n* = 8).

Table S1. Demographic data of healthy donors (HDs) and patients with multiple myeloma.

	**HD**	**MM**	***P*-value**
Number of subjects	16	10	
Median age	31.1 (22.0 - 40.0)	58.6 (47.0 - 69.0)	<0.0001
Gender (Female/Male)	10/6	4/6	0.4216
NK cell (%)	10.9 (2.95 - 28.7)	14.3 (1.53 - 22.2)	0.12

*Values are presented as the median (range).

Table S2. Individual characteristics of 10 patients with multiple myeloma.

Patient	**MM1**	**MM2**	**MM3**	**MM4**	**MM5**	**MM6**	**MM7**	**MM8**	**MM9**	**MM10**
Age	65	69	58	47	54	54	67	69	57	56
Gender	Female	Male	Male	Male	Female	Male	Female	Female	Male	Male
Disease setting	Newly diagnosed MM	Relapsed or refractory MM	Relapsed or refractory MM	Relapsed or refractory MM	Newly diagnosed MM	Relapsed or refractory MM	Relapsed or refractory MM	Relapsed or refractory MM	Relapsed or refractory MM	Newly diagnosed MM
ISS	1	2	1	2	1	1	2	3	2	3
R-ISS	1	2	1	2	unknown	1	2	2	2	3
Hb (g/dL)	13.8	15.3	14.4	12.4	11	14.2	9.2	10.3	15	8.7
WBC (10^3/uL)	4.2	4.5	6.8	4.2	4.2	8.8	2.3	3.4	7.8	3.2
Serum β2 microglobulin (μg/mL)	2.5	3.1	1.7	1.3	2.5	1.7	3.6	7	1.9	33
Serum LDH (IU/L)	199	238	258	120	161	222	757	192	202	389
Serum albumin (g/dL)	3.6	3.6	4	3.4	3.6	4	3.3	3.8	3.6	4.3
Serum M-protein (g/dL)	1	1.6	0	0.2	0.6	0	0.1	0	0.4	1.5
Urine M-protein (mg/day)	-	0	0	0	0	0	931	77.8	0	3145
Myeloma subtype	IgG	IgG	Light chain only	IgG	IgA	Light chain only	IgA	Light chain only	IgG	IgD
Isotype	κ	λ	κ	κ	λ	λ	λ	κ	λ	λ
Serum level of IgG (mg/dL)	-	2070	577	675	1020	1570	545	882	1110	337

ISS: International staging system, R-ISS: Revised-international staging system
